# Pro-inflammatory diet in pregnancy associated with increased risk of abnormal perinatal outcomes: evidence from dietary inflammatory index in the UK biobank

**DOI:** 10.3389/fpubh.2026.1819828

**Published:** 2026-08-11

**Authors:** Lili Sheng, Wei He, Fang Wang, Xiaochen Weng, Mingjing Zhou, Shengnan Wu, Weiming Cao, Ye Luo, Kunming Li

**Affiliations:** 1 Department of Gynecology, Shanghai Key Laboratory of Maternal Fetal Medicine, Shanghai Institute of Maternal Fetal Medicine and Gynecologic Oncology, Shanghai First Maternity and Infant Hospital, School of Medicine, Tongji University, Shanghai, China; 2 School of Public Health, Fudan University, Shanghai, China; 3 Department of Gynecology, Shanghai East Hospital, School of Medicine, Tongji University, Shanghai, China; 4 Department of Integrated Traditional Chinese Medicine (TCM) and Western Medicine, Shanghai Key Laboratory of Maternal Fetal Medicine, Shanghal Institute of Maternal Fetal Medicine and Gynecologic Oncology, Shanghai First Maternity and Infant Hospital, School of Medicine, Tongji University, Shanghai, China; 5 Shanghai Health and Medical Center (SHMC), Jiangsu, China; 6 Yunnan Provincial Health and Rehabilitation Center, Yunnan, China; 7 TCM Development Office (Education and Research Office), Punan Branch of Renji Hospital, Shanghai Jiaotong University School of Medicine (Punan Hospital in Pudong New District, Shanghai), Shanghai, China; 8 Department of Reproductive Medicine, Shanghai Tenth People's Hospital, Tongji University School of Medicine, Shanghai, China

**Keywords:** abnormal perinatal outcomes, dietary intake, DII (dietary inflammatory index), pro-inflammatory diet, UK biobank

## Abstract

**Objectives:**

This study aimed to investigate the association between a pro-inflammatory diet in pregnancy, assessed *via* the Dietary Inflammatory Index (DII), and abnormal perinatal outcomes (APOs), including miscarriage and stillbirth. Leveraging the UK Biobank cohort, we evaluated whether higher DII scores were associated with increased APOs risk. Additionally, we explored interactions between DII, lifestyle factors (physical inactivity, smoking exposure), and socioeconomic determinants to identify modifiable pathways for reducing APOs.

**Methods:**

We analyzed 10,353 women from the UK Biobank with pregnancy records and ≥2 valid 24-h dietary assessments (collected during pregnancy between 2006 and 2019). DII scores were computed using intakes of 20 nutrients (e.g., fats, vitamins, and fiber) linked to inflammation. APOs (miscarriage/stillbirth) were derived from self-reported questionnaires. Covariates (age, BMI, physical activity, income, education, and smoking) were adjusted in logistic regression models comparing DII quartiles (Q1: anti-inflammatory; Q4: pro-inflammatory). A sensitivity analysis using miscarriage and stillbirth as separate outcomes was conducted. A formal test for nonlinear trend (p-for-trend) was performed across quartiles. Analyses used R 4.1.1 and Python 3.7.

**Results:**

Compared with the most pro-inflammatory group (Q4), the risk of abnormal perinatal outcomes was 14% lower in the most anti-inflammatory group (Q1; OR = 0.86, 95% CI: 0.76–0.97) in model 1, 13% lower (OR = 0.87, 95% CI: 0.77–0.98) in model 2, and 14% lower (OR = 0.86, 95% CI: 0.76–0.97) in model 3. Additionally, compared with the most pro-inflammatory group (Q4), the low anti-inflammatory group (Q2) was associated with an 17% lower risk of abnormal perinatal outcomes in model 3 and low pro-inflammatory group (Q3) was associated with an 12% lower risk of abnormal perinatal outcomes in model 3. Physically inactive women, smokers, and those with lower education had higher DII scores and APO incidence (*p* < 0.001). Household income and tobacco exposure were also significantly associated with APOs (*p* < 0.05).

**Conclusions:**

Higher DII scores were associated with higher risks of miscarriage and stillbirth. Anti-inflammatory diets confer protective effects, underscoring diet optimization as a key intervention strategy. Lifestyle factors (inactivity, smoking) and lower education amplify both pro-inflammatory diets and APO susceptibility. These findings support integrating dietary inflammation screening into prenatal care to mitigate adverse outcomes.

## 1 Introduction

The definition of adverse perinatal outcomes (APOs) typically encompasses a range of conditions that adversely affect neonatal health, which may manifest immediately at birth or gradually develop over a period after delivery. APOs primarily include preterm birth, low birth weight, neonatal asphyxia, congenital malformations, miscarriage, stillbirth, and neonatal death. In a previous study, they define APOs as preterm delivery, small for gestational age, preeclampsia, other hypertensive disorders, and gestational diabetes (1). Their data indicate that approximately 30% of women of reproductive age experience APOs (1). These outcomes not only impact short-term maternal and neonatal health but may also have long-term adverse effects on offspring development. Therefore, it is crucial to study how to prevent APOs.

During a normal pregnancy, the maternal body undergoes physiological adaptations to maintain health and support fetal development, including immune system modulation (2). A balanced inflammatory response is critical in this process (3). This specialized state is characterized by a shift in the balance of immune cells and signaling molecules, prominently featuring: an upregulation of anti-inflammatory components, such as regulatory T cells (Tregs) and the cytokines Interleukin-10 (IL-10) and Transforming Growth Factor-beta (TGF-β), which actively suppress detrimental immune responses against paternal antigens present in the fetus (4–6). A controlled pro-inflammatory environment remains essential for processes like trophoblast invasion and parturition (7, 8), and the maintenance of this precise balance is fundamental for successful gestation. A disruption of this equilibrium, particularly a shift toward a dominant pro-inflammatory state, creates a hostile gestational environment. This imbalance can contribute to the pathogenesis of APOs, such as pre-eclampsia, preterm birth, and fetal growth restriction, by promoting endothelial dysfunction, excessive immune activation, and impaired placental development (7–10).

Since this inflammatory balance is critical for successful pregnancy and its dysregulation is one of the core mechanisms underlying APOs, identifying key modulators for routine intervention is of paramount importance (11, 12). Among numerous environmental and behavioral factors, maternal dietary patterns are the preferred entry point due to their direct, sustained, and systemic impact on systemic inflammatory status. Various nutrients and bioactive substances in the diet can modulate key inflammatory signaling pathways, thereby influencing the levels of pro-inflammatory and anti-inflammatory cytokines (13). In the same time, previous studies have demonstrated that dietary patterns differentially influence immune responses. For example, the Mediterranean diet exhibits anti-inflammatory effects, whereas Western diets are pro-inflammatory (14). Consequently, quantitatively assessing the inflammatory potential of the overall diet becomes a critical entry point for predicting and intervening in pregnancy-related inflammatory dysregulation. To this end, this study employs the Dietary Inflammation Index (DII) as a comprehensive quantitative tool to investigate how maternal daily dietary patterns during pregnancy affect the risk of APOs by modulating this core aspect of inflammatory balance (15). Current research has partially explored the potential moderating roles of lifestyle factors and socioeconomic factors such as household income and education level in this association, yet significant gaps remain (16, 17).

There is growing evidence linking DII to APOs such as preterm birth and gestational diabetes mellitus (18); however, no large-scale prospective study has specifically investigated the relationship between DII and pregnancy loss (including miscarriage and stillbirth) (19). Furthermore, the potential moderating roles of lifestyle factors and socioeconomic factors in this association require further investigation. This study focuses on miscarriage and stillbirth as primary indicators of APOs, analyzing them from both composite outcome and independent endpoint perspectives, as both represent forms of pregnancy loss sharing common inflammatory pathophysiological mechanisms. Consequently, we aimed to examine the association between DII and APOs (miscarriage and stillbirth) within the UK Biobank cohort, proposing the hypothesis that higher DII scores are associated with increased risk of APOs, while lifestyle and socioeconomic factors may modulate this relationship.

## 2 Methods

### 2.1 Study population

The UK Biobank is a prospective population-based cohort study that recruited half a million participants from the general population between 2006 and 2010 (20). The UK Biobank protocol is available online (http://www.ukbiobank.ac.uk/wp-content/uploads/2011/11/UK-Biobank-Protocol.pdf). Participants had to be registered with a general practitioner and live within 25 miles of an assessment center (England, Wales, and Scotland) to take part. Participants provided extensive information through questionnaires, interviews, health records, physical measurements, and samples of blood and urine. The UK Biobank Study was approved by the North West Multi-Center Research Ethics Committee (REC reference for UK Biobank 11/NW/0274). All participants provided informed consent electronically by signing on a touchscreen using a signature-capture device.

In the UK Biobank, participants underwent four dietary questionnaires between 2006 and 2019. Those who had completed at least two web-based 24-h dietary assessments were included in the analysis (*n* = 126,855). We excluded participants with invalid total energy estimated intake data (<1,200 or >3,500 kcal/day for women) and those without any pregnancy-related records. A total of 10,353 participants were finally included in our study. The mean age of participants at dietary recall was approximately 57 years. And the median gestation was not available in the UK Biobank dataset. Dietary assessment was conducted after pregnancy onset. We conducted this study assuming that dietary patterns remained largely unchanged throughout the pre-pregnancy period, during pregnancy, and postpartum; however, it must be acknowledged that this assumption may introduce potential bias.

### 2.2 Assessment of dietary intake

In the UK Biobank, dietary information was collected using the Oxford WebQ, a web-based self-administered 24-h dietary assessment questionnaire developed for using in large population studies. In this study, all questionnaires (the Oxford WebQ) completed by the participants were conducted during pregnancy. UK Biobank participants were invited up to five times to complete the Oxford WebQ dietary assessment, and we calculated mean values from the available data. The Oxford WebQ dietary assessment asks about the consumption of over 200 common food and beverage items in the previous 24 h (21). We created new variables for the weight (gram) from the 24-h dietary assessments to estimate the daily intake of each food or drink. The top frequency category was open-ended so we coded 3 + = 3, 4 + = 4, 5 + = 5, 6 + = 6 and less than one was coded as 0.5. Weight was generated by multiplying the frequency of consumption by the serving size in grams. Then, we used McCance and Widdowson's the Composition of Foods Integrated Dataset (Version 2021) to calculate participants' nutrient intakes based on food weights, in order to further compute DII.

### 2.3 Calculation of dietary inflammatory index

The DII is a review of thousands of articles discussing the effects of 45 dietary components on inflammation (22). A greater pro-inflammatory effect reflects a higher score, while a greater anti-inflammatory effect is represented by a lower score (22). When calculating the DII, we included 20 nutrients, namely: carbohydrate, energy, fiber, folate, saturated fatty acids, polyunsaturated fatty acids, monounsaturated fatty acids, protein, fat, vitamin B6, vitamin B12, magnesium, iron, vitamin C, vitamin E, cholesterol, zinc, selenium, vitamin D, thiamin. Even though the number of nutrients used for the calculation of DII was less than 30, the DII scores were still available. The specific DII calculation methods have been previously described (23).

### 2.4 Assessment of abnormal perinatal outcomes

In this study, we focus on miscarriage, stillbirth, and other complications as our primary APOs of interest. Miscarriage was defined as spontaneous pregnancy loss before 20–24 weeks' gestation, consistent with the UK Biobank definition. We grouped miscarriage and stillbirth together for the main analysis because both represent pregnancy loss and share potential inflammatory pathophysiology, allowing for greater statistical power to detect an overall association with DII. We acknowledge the etiological differences and therefore conducted additional sensitivity analyses for each outcome separately. In the UK Biobank, participants were asked, “Ever had stillbirth, spontaneous miscarriage, or termination?” and “Number of spontaneous miscarriages.” All participants provided quantities for each type. Based on the answers to these two questions, we excluded individuals with no response records and those with contradictory answers, ultimately deriving participants' abnormal perinatal outcomes.

### 2.5 Assessment of baseline covariates

In the UK Biobank, the baseline questionnaire was used to assess several possible confounding variables including age, physical activity, BMI, household income, whether alcohol drinker, education level, whether they are currently smoker or exposed to secondhand smoke. Physical activity metabolic equivalents <10 Met-h per week was considered inactive, whereas activity at or above this threshold was classified as active. Alcohol drinker was defined as any current drinking at the time of the baseline questionnaire. Household income was measured in British pounds. BMI was categorized using standard World Health Organization cutoffs for European populations: normal (18.5–24.9), underweight (<18.5), overweight (25.0–29.9), and obese (≥30.0). However, these measurements may not have been taken during pregnancy. It must be acknowledged that this has limitations.

### 2.6 Statistical analysis

The differences in characteristics between participants with and without abnormal perinatal outcomes, as well as the distribution of DII across characteristics, were examined using the chi-squared test for proportional variables. Using the highest DII score group (Q4, most pro-inflammatory group) as the reference group, we used logistic regression to assess the risk of abnormal perinatal outcomes in populations with DII scores in Q1 (most anti-inflammatory group), Q2 (low anti-inflammatory group), and Q3 (low pro-inflammatory group). In the logistic regression analysis: model 1 did not adjust for any covariates; model 2 adjusted for age, physical activity, household income, and smoke exposure; model 3 additionally adjusted for BMI, alcohol consumption, and education on the basis of model 2. Sensitivity analyses were performed using miscarriage alone and stillbirth alone as outcomes. A formal test for nonlinear trend (*p*-for-trend) was performed across quartiles by entering the median DII value of each quartile as a continuous variable in the regression model. All data processing and statistical analyses were performed using R version 4.1.1 (Auckland, New Zealand) and Python version 3.7 (Wilmington, DE, USA).

## 3 Results

Table 1 describes the distribution of several characteristics by the occurrence of abnormal perinatal outcomes. Women with abnormal perinatal outcomes were more likely to have the following characteristics: younger than 50 years or older than 60 years, inactive physical activity, higher household income, and tobacco exposure (current smoking or frequent secondhand smoke exposure).

**Table 1:** Baseline characteristics of women in the UK biobank cohort (included records of perinatal outcomes and valid dietary survey data) by occurrence of abnormal perinatal outcomes.

Character	Total (*N* = 10,353)	Abnormal perinatal outcomes	Non-abnormal perinatal outcomes	*P*
Age				0.002
<50	1,397 (13.49%)	454 (32.50%)	943 (67.50%)	
50~60	2,974 (28.73%)	866 (29.12%)	2,108 (70.88%)	
>60	5,982 (57.78%)	1,963 (32.82%)	4,019 (67.18%)	
Physical activity				<0.001
active	5,709 (55.14%)	1,241 (21.74%)	4,468 (78.26%)	
inactive	4,644 (44.86%)	2,042 (43.97%)	2,602 (56.03%)	
BMI				0.469
Normal weight	4,581 (44.25%)	1,425 (31.11%)	3,156 (68.89%)	
Overweight	3,516 (33.96%)	1,138 (32.37%)	2,378 (67.63%)	
Obesity	2,256 (21.79%)	720 (31.91%)	1,536 (68.09%)	
Household income				0.025
<18,000	2,135 (20.62%)	655 (30.68%)	1,480 (69.32%)	
18,000~30,999	2,777 (26.82%)	831 (29.92%)	1,946 (70.08%)	
31,000~51,999	2,754 (26.60%)	907 (32.93%)	1,847 (67.07%)	
52,000~100,000	2,033 (19.64%)	660 (32.46%)	1,373 (67.54%)	
>100,000	654 (6.32%)	230 (35.17%)	424 (64.83%)	
Alcohol drinker				0.183
Yes	9,285 (89.68%)	2,964 (31.92%)	6,321 (68.08%)	
No	1,068 (10.32%)	319 (29.87%)	749 (70.13%)	
Education level				0.404
GCSE or less	4,551 (43.96%)	1,442 (31.69%)	3,109 (68.31%)	
Prof qual/HND/A levels	1,485 (14.34%)	492 (33.13%)	993 (66.87%)	
College/University degree	4,317 (41.70%)	1,349 (31.25%)	2,968 (68.75%)	
Smoke exposure				<0.001
Yes	4,982 (48.12%)	2,283 (45.82%)	2,699 (54.18%)	
No	5,371 (51.88%)	1,000 (18.62%)	4,371 (81.38%)	

Table 2 describes the distribution of characteristics across quartiles of DII scores. Women in the most pro-inflammatory group (quartile 4) were more likely to have the following characteristics: younger than 60 years, inactive physical activity, non-current drinkers, lower educational attainment (GCSE or less), current smoking, or frequent secondhand smoke exposure. BMI and household income showed no significant differences across DII quartiles. Therefore, it is not displayed in the table.

**Table 2:** Baseline characteristics of women in the UK biobank cohort (included records of perinatal outcomes and valid dietary survey data) according to quartiles of DII.

Character	Q1	Q2	Q3	Q4	*P*
Age					<0.001
<50	331 (23.69%)	328 (23.48%)	376 (26.91%)	362 (25.91%)	
50~60	1,440 (24.07%)	1,450 (24.24%)	1,504 (25.14%)	1,588 (26.55%)	
>60	817 (27.47%)	810 (27.24%)	708 (23.81%)	639 (21.49%)	
Physical activity					<0.001
active	1,128 (29.35%)	973 (25.32%)	928 (24.15%)	814 (21.18%)	
inactive	1,460 (22.43%)	1,615 (24.81%)	1,660 (25.50%)	1,775 (27.27%)	
Alcohol drinker					0.026
Yes	2,351 (25.32%)	2,325 (25.04%)	2,323 (25.02%)	2,286 (24.62%)	
No	237 (22.19%)	263 (24.63%)	265 (24.81%)	303 (28.37%)	
Education level					<0.001
GCSE or less	1,023 (22.48%)	1,080 (23.73%)	1,142 (25.09%)	1,306 (28.70%)	
Prof qual/HND/A levels	408 (27.47%)	357 (24.04%)	371 (24.98%)	349 (23.50%)	
College/University degree	1,157 (26.80%)	1,151 (26.66%)	1,075 (24.90%)	934 (21.64%)	
Smoke exposure					0.001
Yes	1,614 (24.26%)	1,619 (24.33%)	1,694 (25.46%)	1,727 (25.95%)	
No	974 (26.33%)	969 (26.20%)	894 (24.17%)	862 (23.30%)	

Compared with the most pro-inflammatory group (Q4), the risk of abnormal perinatal outcomes was 14% lower in the most anti-inflammatory group (Q1; OR = 0.86, 95% CI: 0.76–0.97) in model 1, 13% lower (OR = 0.87, 95% CI: 0.77–0.98) in model 2, and 14% lower (OR = 0.86, 95% CI: 0.76–0.97) in model 3 (Table 3). Notably, the low anti-inflammatory group (Q2) showed the strongest protective association, with a 17% lower risk of abnormal perinatal outcomes in model 3 (Table 3). The low pro-inflammatory group (Q3) was associated with an 12% lower risk of abnormal perinatal outcomes in model 3 (Table 3). Sensitivity analyses examining miscarriage and stillbirth separately yielded results consistent in direction and magnitude with the composite outcome analysis (data not shown in main tables for brevity).

**Table 3:** Relative risks of abnormal perinatal outcomes for associations between different DII quartiles.

DII	Model 1^a^			Model 2^b^			Model 3^c^		
	OR	*P*	*P*-trend	OR	*P*	P-trend	OR	*P*	P-trend
Q1	0.86 (0.76,0.96)	0.009	0.0258	0.87 (0.77,0.98)	0.019	0.0295	0.86 (0.76,0.97)	0.012	0.0301
Q2	0.82 (0.73,0.92)	0.001		0.83 (0.74,0.93)	0.002		0.83 (0.73,0.93)	0.001	
Q3	0.88 (0.79,0.99)	0.037		0.89 (0.79,1.00)	0.041		0.88 (0.79,0.99)	0.037	
Q4	Reference			Reference			Reference		

^a^Model 1 did not adjust for any covariates. ^b^Model 2 adjusted for age, physical activity, household income, and smoke exposure. ^c^Model 3 additionally adjusted for BMI, alcohol consumption, and education on the basis of Model 2.

## 4 Discussion

To our best knowledge, this study is the first to evaluate the relationship between DII and APOs such as miscarriage and stillbirth, demonstrating that higher DII scores are associated with an increased risk of APOs. A total of 10,353 participants were finally included in our study. Key findings include: women with physical inactivity or higher smoke exposure had elevated DII scores and a higher incidence of APOs. After adjusting for covariates, high DII remained directly associated with increased APO risk. An interesting observation was that the strongest protective effect was observed in Q2 rather than Q1. This pattern suggests a potential non-monotonic or U-shaped relationship between the anti-inflammatory potential of diet and APO risk, where a moderately anti-inflammatory diet (Q2) might offer optimal protection, while an extremely anti-inflammatory diet (Q1) might confer slightly less benefit in this cohort. Several possibilities could explain this observation. Biologically, while a strong anti-inflammatory state is generally beneficial, an extremely suppressed inflammatory milieu might theoretically impair necessary inflammatory processes for normal placental development and remodeling. Alternatively, this could reflect measurement nuances; individuals in Q1 might have dietary patterns that, while scoring as highly anti-inflammatory based on the DII algorithm, lack other essential nutrients not fully captured by the index, or their reported intake might be subject to greater measurement error. Residual confounding by unmeasured lifestyle or socioeconomic factors differentially distributed across the extreme quartiles is also a possibility. This finding warrants further investigation in future studies with more detailed dietary and physiological assessments.

The association between DII and APOs involves multiple biological mechanisms, including inflammatory response, immune regulation and oxidative stress (24–26). A high-DII diet may induce systemic inflammatory response, affect placental function, maternal-fetal nutrient exchange, and vascular function, and ultimately lead to APOs (27). Firstly, high DII may disrupt early maternal-fetal immune tolerance (15, 28). Successful pregnancy establishment depends on maternal immune tolerance to the embryo (29). The pro-inflammatory state promoted by a high DII diet may inhibit the differentiation and function of regulatory T cells in the uterine decidual tissue, while simultaneously overactivating cytotoxic phenotypes such as decidual natural killer cells (12, 28). This imbalance can directly attack trophoblast cells or interfere with their normal invasion, resulting in implantation failure or early miscarriage. Secondly, high DII may impair early placental development and vascular remodeling. Successful remodeling of uterine spiral arteries by trophoblasts during early pregnancy is fundamental to ensuring placental blood supply (30). Excessive inflammation and oxidative stress associated with high DII can disrupt trophoblast proliferation, invasive capacity, and endothelial function, leading to insufficient spiral artery remodeling and poor placental perfusion, which may cause embryonic developmental arrest or miscarriage (26, 30). Finally, high DII may induce a thrombotic state and intravascular dysfunction (31). Inflammatory factors [e.g., Interleukin-6 (IL-6), Tumor Necrosis Factor-alpha (TNF-α)] associated with high DII can activate vascular endothelium, promote adhesion molecule expression and procoagulant substance release, creating a thrombotic state in placental microvessels (31). These microthrombi can obstruct early embryonic blood supply, representing a critical mechanism for pregnancy loss (particularly stillbirth) (31).

Pregnant women who are physically inactive have a higher risk of adverse perinatal outcomes. However, previous studies have yielded inconsistent results regarding the relationship between physical activity during pregnancy and adverse outcomes. Research has found that engaging in physical exercise during mid-pregnancy is associated with adverse perinatal outcomes (32); a similar study has also found that consistently low levels of physical activity are linked to an increased risk of APOs (32, 33). These findings may suggest that the relationship between physical activity and pregnancy outcomes may be time-and intensity-dependent. Moderate activity during early pregnancy may lay a favorable foundation for gestation by improving basal metabolism and an anti-inflammatory environment, whereas inappropriate high-intensity exercise in mid-pregnancy may induce physiological stress in certain individuals. Exposure to environmental tobacco smoke adversely affected pregnancy outcomes, consistent with earlier research. A Korean study showed that compared with the smoke-free exposure group, the AOR of preterm birth was 1.7 and the AOR of low birth weight was 2.3 in the smoke-exposed group (34).

Physical inactivity, lower education levels, and higher smoke exposure were linked to higher DII scores. Physical activity may modulate inflammatory responses, with higher activity levels potentially reducing DII (35). Individuals with higher education levels tend to have greater awareness of healthy dietary practices and are more likely to choose anti-inflammatory foods, thereby lowering their DII (36). But higher household income has a higher association with incidence of APOs in our study. It is contradictory to the findings. This unexpected result may be influenced by residual confounding factors, such as unmeasured occupational stress, healthcare-seeking behaviors, or other lifestyle factors not fully captured in our models. Exposure to secondhand smoke can activate the immune system, triggering inflammatory mediators that may amplify the pro-inflammatory effects of diet (37).

Our results demonstrated that a low-DII dietary pattern was associated with a lower risk of APOs compared to a high-DII pattern. This is consistent with previous studies. A case-control study included 67 incident cases and 68 controls among Iranian women found a correlation between pro-inflammatory diets and miscarriage risk. The results showed that the high DII group had a higher risk of miscarriage (OR = 2.12, 95% CI: 1.02–4.43) (38). In addition, a study has also shown associations between high DII and APOs, suggesting that inflammatory factors may influence pregnancy outcomes through epigenetic modifications. A study in the United States selected some of the women in the sample and explored associations between E-DII, circulating cytokines (*n* = 105), and offspring methylation (*n* = 338) as potential modulators of these relationships. The adjusted regression model showed that women with pro-inflammatory diets had a higher rate of preterm birth in their female offspring (β = −0.22), but the effect on male offspring was not significant (27). Additionally, a study in Iran included 122 women with gestational diabetes and 266 controls hospitalized for acute non-tumor diseases. Dietary assessments were made using DII, and those with higher DII scores had a higher risk of developing gestational diabetes mellitus (GDM). The results showed that patients with high DII had a higher risk of GDM (OR = 2.10, 95% CI: 1.02–4.34) (39).

There are several strengths in this study. First, it features a large sample size. A total of 10,353 participants were finally included in our study. Second, it links diet, inflammation, and APOs, conducting detailed subgroup analyses that consider factors such as physical activity intensity, family income, education level, and exposure to smoke. Third, it follows pregnant women for up to 13 years, with up to five follow-up visits. However, several limitation should be acknowledged. First, reverse causality cannot be ruled out. Although we included only dietary assessments completed during pregnancy, the exact timing of these assessments relative to the onset of APOs is not precisely defined. Subclinical pregnancy complications occurring in early pregnancy (such as early placental dysfunction) could affect appetite, food choices, and activity levels, potentially leading to an elevated DII score or reported lower physical activity. Future studies with repeated dietary assessments timed before and after pregnancy onset are needed to strengthen causal inference. Second, the DII used in this study was based on 20 nutrients rather than the full 45-component version. While this simplified DII has been validated and is widely used, it may not capture the full inflammatory potential of the diet. Additionally, the DII reflects the influence of dietary patterns on six classic inflammation markers but does not account for other inflammatory factors and cells. In the same time, each follow-up covers only the past 24 h, which may introduce systematic errors and recall bias. Despite considering many known confounding factors, some potential factors affecting the correlation have not been fully excluded, such as the level of healthcare services in the area of residence, whether there was a mental illness before pregnancy, family history, and any APOs prior to pregnancy. Despite these limitations, the study provides valuable insights for maternal dietary recommendations to reduce APOs risks. First, a questionnaire survey can be employed, using DII as an indicator for dietary inflammation assessment and incorporating it into early prenatal care to identify pregnant women at high risk of miscarriage or stillbirth. Furthermore, dietary recommendations can be provided for high-risk populations. Secondly, relevant public health policies should advocate for an anti-inflammatory diet during pregnancy, particularly those with lower educational attainment or tobacco exposure, who appear to have both higher DII scores and greater APOs susceptibility. Finally, I believe future research should evaluate the efficacy of anti-inflammatory dietary interventions in pregnant women with high DII levels.

## 5 Conclusion

Our study shows that pregnant women with high DII are at a significantly higher risk of APOs, and an anti-inflammatory diet provides protective benefits. Therefore, optimizing the diet to reduce the DII index could be an important intervention strategy to prevent APOs. Our findings also highlight that physical inactivity, tobacco exposure, and lower educational attainment were linked to both higher DII scores and APOs, suggesting lifestyle-inflammatory interactions.

## Data Availability

The datasets presented in this study can be found in online repositories. The names of the repository/repositories and accession number(s) can be found in the article/supplementary material.
